# MiDNE a tool for Multi-omics genes and drugs interactions discovery

**DOI:** 10.1016/j.csbj.2025.10.022

**Published:** 2025-10-15

**Authors:** Aurora Brandi, Barbara Majello, Ines Simeone, Massimiliano Romano, Michele Ceccarelli, Giovanni Scala

**Affiliations:** a Department of Biology, University of Naples “Federico II”, Via Vicinale Cupa Cintia 26, Naples, 80126, Italy; b Department of Electrical Engineering and Information Technologies, University of Naples “Federico II”, Via Claudio 21, Naples, 80121, Italy; c Sylvester Comprehensive Cancer Center, University of Miami, Miami, 33131, FL, USA

**Keywords:** Multi-omics data integration, Drug discovery, Cancer research, Systems biology, R package

## Abstract

The availability of models representing molecular interactions in complex pathologies is essential for understanding their molecular setup and identifying therapeutic vulnerabilities. In this context, the advent of high-throughput technologies has enabled the rapid and cost-effective profiling of multiple omics layers, driving a paradigm shift from generalized models to disease-specific, context-aware modeling approaches. While the analysis of individual omics layers can provide information about specific aspects of cellular biology for a given disease, it often fails to capture complex interactions among molecules and drugs operating across different regulatory levels. Here, we introduce MiDNE (Multi-omics genes and Drugs Network Embedding), a novel computational framework that integrates experimental multi-omics data with pharmacological knowledge to uncover disease specific multi-omics gene and drug interactions. MiDNE integrates omics-specific networks, derived from experimental data, with known drug interactors in a multiplex heterogeneous network. It applies a network embedding procedure based on the random walk with restart algorithm to project genes and drugs into a shared multi-omics latent space, enabling gene–drug clustering and neighborhood search. We demonstrate the potential of MiDNE on Breast Invasive Carcinoma and Glioblastoma multiforme, by integrating gene expression, methylation, proteomic, and copy number variation profiles with curated drug–target interactions. By providing multilayer and disease-specific views of gene and drug interactions, MiDNE facilitates the discovery of actionable gene–drug relationships and the development of precision pharmacological strategies. MiDNE is available as both an open-source R package and a Shiny web application.

## Highlights

• MiDNE is a novel R package for integrating gene-centered multi-omics and drug data.
• MiDNE allows the discovery of gene–gene, drug–gene, and drug–drug associations from cancer derived omics data.
• MiDNE offers a user-friendly Shiny interface enables easy execution and exploration of its pipeline.
• MiDNE has been benchmarked using BRCA and GBM multi-omics data and FDA-approved drugs.
• MiDNE provides a network-based tool for cancer research and drug repurposing.

## Graphical Abstract

**Figure:**
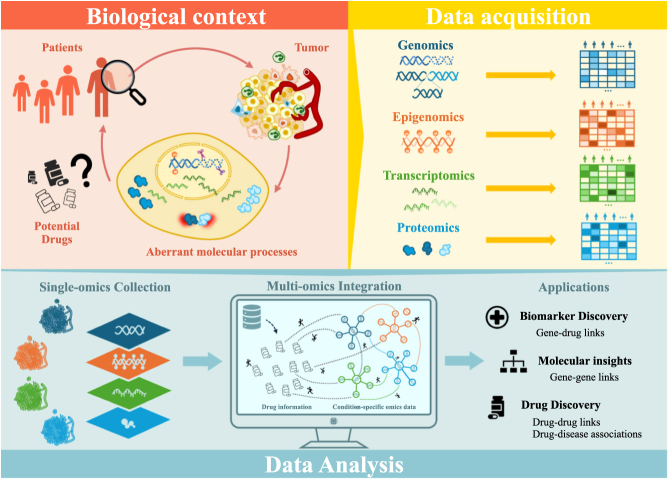

**Abbreviation**
BRCA: Breast invasive Carcinoma
CDAP: Common Data Analysis Pipelines
CNV: Copy Number Variation
CPTAC: Clinical Proteomic Tumor Analysis Consortium
DBSCAN: Density-Based Spatial Clustering of Applications with Noise
DTINet: Drug–Target Interaction Network
FDA: Food and Drug Administration
GBM: Glioblastoma Multiforme
GO: Gene Ontology
GRN: Gene Regulatory Network
KEGG: Kyoto Encyclopedia of Genes and Genomes
MH: Multiplex Heterogeneous
MiDNE: Multi-omics genes and Drugs Network Embedding
MOFA: Multi-Omics Factor Analysis
PCA: Principal Component Analysis
RPKM: Reads Per Kilobase Million
RWRA: Random Walk with Restart Algorithm
SMPDB: Small Molecule Pathway Database
TCGA: The Cancer Genome Atlas
t-SNE: t-distributed Stochastic Neighbor Embedding
UMAP: Uniform Manifold Approximation and Projection

## 1 Introduction

The construction of biologically meaningful systems biology models represents a challenging task due to the inherent complexity and heterogeneity of molecular interactions occurring within and among different molecular districts [1]. In this context, the integration of data from different omics layers is instrumental in achieving a deeper understanding of the underlying molecular landscape of a given disease and increasing the predictive power for the system’s response to external factors [2]. In cancer research, multi-omics data integration has classically been used to create multi-omics molecular profiles of samples and to perform subtyping [3], [4], [5]. On the other hand, multi-omics gene-centered models are particularly valuable in oncology research. By enabling a multi-layered dissection of the tumor system, these latter not only help uncover key aberrations underlying the pathological phenotype, but can also guide the design of targeted therapies aimed at disrupting cancer progression [6]. However, gene-based integration models face several challenges, including the intrinsic sparsity of omics data resulting from experimental variability and batch effects as well as the high level of noise characteristic of large-scale measurements, which can obscure biologically meaningful signals [7]. Moreover, the biological and technical heterogeneity across omics layers further complicates integration [7]. Intermediate integration methods offer an effective strategy to address these limitations. Unlike early integration approaches, they are less sensitive to semantic heterogeneity among omics layers and can preserve both intra- and inter-layer dependencies [2]. These methods typically transform input matrices into a shared intermediate representation before integration. Such representations can take the form of latent spaces — as in methods like Non-negative Matrix Factorization or Multi-Omics Factor Analysis (MOFA) [4] — or networks, which are particularly well suited for modeling interactions among molecular entities, since a network represents nodes connected by edges that encode their statistical or functional associations [8], [9], [10]. Network-based models, such as co-expression networks [11], gene regulatory networks (GRNs) [12], and protein–protein interaction (PPI) networks [13], have been successfully employed in systems biology to model gene associations derived from transcriptomics and protein interaction data across various diseases [14]. In contrast, only a limited number of studies have explored the use of other molecular layers, such as DNA methylation and genomic variation [15], [16], [17], for constructing disease-specific gene networks.

A type of network that effectively captures the complex organization of biological systems is the Multiplex network, as it preserves the distinct identity of each omics layer while simultaneously modeling inter-layer connections. Such a structure can be seamlessly extended to include relationships with additional biological entities (such as diseases [18], metabolites, or drugs [19]) that are often difficult to integrate using alternative approaches, thereby giving rise to a Multiplex Heterogeneous (MH) network. The multi-view information encoded in these networks can be effectively extracted using diffusion-based methods, such as the Random Walk with Restart Algorithm (RWRA) [20]. RWRA is particularly advantageous in this context due to its ability to preserve both local and global latent structures within the integrated networks, its reliance on only a few intuitive parameters, and its capacity to produce easily interpretable results. For instance, AMEND [15] employs RWRA as a layer embedding method to extract core sub-modules from a MH network by prioritizing differentially expressed genes between two conditions; RandomWalkRestart [18] applies RWRA as a node embedding strategy on a MH network composed of genes and diseases to identify hallmark genes for various pathologies; and DTINet [21] leverages RWRA to independently compute diffusion profiles for proteins and drugs, which are subsequently embedded into a low-dimensional space to infer drug–target associations. However, several shortcomings can be identified in existing gene-based integration methods. For example, AMEND, RandomWalkRestart, and DTINet, while providing efficient RWRA-based integration strategies, do not include procedures for network inference. In fact, DTINet relies only on prior knowledge networks and only predicts drug–target interactions and AMEND requires input data from two matched conditions to extract sub-networks.

Here, we present *Multi-omics genes and Drugs Network Embedding* (MiDNE), a network-based framework that integrates multi-omics and drug interaction data using a RWRA-based node embedding approach to generate and explore gene-centered models. MiDNE enables the unsupervised discovery of condition-specific multi-omics gene–gene, drug–drug, and drug–gene interactions by analyzing an embedded representation of their integrated network relationships. Specifically, MiDNE: (i) constructs gene networks directly from an arbitrary number of omics matrices (e.g., transcriptomics, proteomics, structural variants and DNA methylation data) using metrics tailored to the intrinsic properties of each omics layer, even in the absence of matched control samples; (ii) integrates heterogeneous features, including genes and drugs, through a RWRA-based embedding strategy; (iii) preserves information from all integrated features within a unified low-dimensional embedding; and (iv) enables the interpretation of captured associations by tracing the contribution of each omics layer and through clustering and enrichment analyses. MiDNE has been benchmarked using multi-omics data from the TCGA and CPTAC data collections on Breast Invasive Carcinoma (BRCA) and Glioblastoma multiforme (GBM) [22], [23] along with known drug–target interactions from the DrugBank database [24]. Its performance was benchmarked against MOFA [4], a well-established multi-omics integration framework, by evaluating their respective abilities to capture gene–gene and drug–gene functional associations. MiDNE is distributed as an R package available on GitHub (https://github.com/BioinfoUninaScala/MiDNE), and as a user-friendly Shiny web application that can be easily launched as a Docker container - requiring no local R installation or configuration.

## 2 Methods

### 2.1 Data collection and processing

MiDNE was benchmarked on a four-omics dataset from Breast Invasive Carcinoma (BRCA) and a three-omics dataset from Glioblastoma Multiforme (GBM). The normalized multi-omics datasets were retrieved from LinkedOmics database [25]. For both tumor types, three omics matrices were considered: (i) gene-level RNA-seq normalized counts, expressed as log(RPKM + 1); (ii) gene-level DNA methylation data from the Infinium Human Methylation 450 K platform, summarized and expressed as β values - 0.5; and (iii) thresholded gene-level somatic copy number variation (CNV) data, expressed as positive, negative, or zero values to indicate the presence of amplifications, deletions, or the absence of indel events, respectively. Additionally, CPTAC mass spectrometry–based proteomic data [23], processed through the Common Data Analysis Pipelines (CDAP), were retrieved and represented as log-ratio expression values for BRCA only, since this omics layer was available exclusively for this tumor type. A constant value of 0.5 was added to all entries of the methylation matrices to obtain the original β values, and the missing values were then imputed using the median value for each gene across samples with available measurements.

Gene–drug associations were retrieved from the "Drug Target Identifiers" list of the DrugBank database (Release Version 5.1.13) [24], restricted to 1795 FDA-approved pharmacologically active drugs and their 940 reported target genes.

To assess MiDNE’s ability to capture condition-specific associations, a generic protein–protein interaction (PPI) network was obtained from the Decagon project [26].

### 2.2 Omics networks inference

MiDNE infers associations between pairs of genes in each omics layer by applying different statistical tests, selected according to the omics under consideration.

Co-expression gene interaction relationships are inferred by evaluating linear correlation between gene expression profiles. In particular, for each possible unordered pair of genes, the Pearson correlation coefficient is computed along with the Pearson correlation test with Benjamin-Hockemberg FDR p-value adjustment. The same criterion is applied to model co-abundance relationships in the proteomic layer, but using the Spearman correlation coefficient along with the Spearman correlation test.

Co-methylation, co-amplification and co-deletion associations are derived by first discretizing the respective omic matrices and then applying an independence test between each pair of genes. In particular, the methylation matrix is discretized into binary values (0/1, unmethylated/methylated) using a user-defined threshold on the β values, while amplification and deletion matrices are derived from CNV matrix as two separate binary matrices, respectively indicating amplification and deletion events. For each matrix obtained in the above passage (methylation, amplification, and deletion), a Fisher’s exact test is performed on all possible unordered pairs of genes based on their binary status. Next, for all gene pairs with a Bonferroni adjusted p-value below a user defined threshold, a post hoc analysis is performed using the chisq.theo.multcomp R function to identify significant co-methylation, co-deletion or co-amplification events.

Formally, starting from L omics matrices, MiDNE generates a set of L undirected gene networks defined as $G_{\alpha }=(V_{\alpha },E_{\alpha })$, with α={1,…,L}. The set of nodes $V_{\alpha }=\{g_{i}^{\alpha },\ldots ,g_{n}^{\alpha }\}$ represents the set of genes profiled in the corresponding omics matrix α. The edge set $E_{\alpha }=\{(g_{i}^{\alpha },g_{j}^{\alpha },w_{i,j}^{\alpha })|\;g_{i}^{\alpha },g_{j}^{\alpha }\in V_{\alpha };\;i\neq j;\;sig_{\alpha }(g_{i},g_{j});\;w_{i,j}^{\alpha }=stat(g_{i}^{\alpha },g_{j}^{\alpha })\}$ includes all the unordered pairs of genes that satisfy the significance criterion $sig_{\alpha }$ defined by the statistical association test for the omics α, with edge weights equal to the value of the corresponding test statistics.

### 2.3 Multiplex omics network

The above defined L omics networks cover the set $Genes=\{\overset{L}{\underset{\alpha =1}{⋃}}label(g)\;|$ $\;g\in V_{\alpha }\}$, where $V_{\alpha }$ is the node set of the network α and label(g) represents the gene ID associated with node g. The single omics networks are then assembled into a Multiplex Omics Network, defined as $G_{M}=(V_{M},E_{M})$, with $V_{M}=\{g_{i}^{\alpha }|\alpha =1,\ldots ,L;\;i=1,\ldots ,|Genes|\}$. This multiplex network is a multi-layer graph, where each layer shares the same node labels (gene IDs) while modeling different types of interactions. Nodes associated with genes that are not originally present in a specific omics - but are present in at least one of the other layers - are added to the corresponding omics layer as isolated nodes; consequently, every gene $g_{i}$ appears in every layer α of $G_{M}$. The set of edges $E_{M}$ contains two types of edges: (i) *intra-layer edges*, reflecting omics-specific associations along with corresponding weights, and (ii) *inter-layer edges*, reflecting inter-omics associations. In this model, inter-layer edges can be defined using two different strategies: (i) in the first strategy, a node $g_{i}$ of the layer α is only connected by an edge with unitary weight to nodes with the same gene ID in the other layers β, with β≠α, while (ii) in the second strategy [27], a node $g_{i}$ is also connected by edges with unitary weight to the projections in each layer β of its first-order neighbor nodes.

### 2.4 Multiplex heterogeneous network

The multi-omics and pharmacological information is assembled into a Multiplex Heterogeneous (MH) network by using the DrugBank drug–gene associations. The latter can be represented as a bipartite graph defined as $DB=(V_{DB}\cup V_{GB},E_{DB})$, where $V_{DB}$ is the set of DrugBank drugs, $V_{GB}\subseteq Genes$ is the subset of genes reported as drug targets in DrugBank, and $E_{DB}=\{(d,g)\;|\;d\in \;V_{DB};\;g\in V_{GB}\}$ is the edge set modeling known drug–gene interactions at the protein level. Consequently, the MH network is modeled as a graph $G_{MH}=(V_{MH},E_{MH})$ where the set of nodes $V_{MH}=V_{M}\cup V_{DB}$, corresponds to the union of the gene nodes in $G_{M}$ and the drug nodes of DB, and the set of edges, $E_{MH}=E_{M}\cup E_{M_{DB}}$, comprises the gene–gene associations as defined in $G_{M}$ and the multiplex gene–drug associations $E_{M_{DB}}=\{(d,g_{\alpha },w_{dg_{\alpha }})|(d,g)\in E_{DB};\;\alpha =1,\ldots ,L;w_{dg_{\alpha }}=1\}$.

### 2.5 Random walk with restart on multiplex heterogeneous network

The integration of multi-omics data and drug information data is performed by encoding the nodes’ neighborhood in the $G_{MH}$ network as probability distribution vectors. An adaptation of the Random Walk with Restart algorithm (RWRA) [18] is applied to all nodes in the $G_{MH}$ network. At each iteration, a node is chosen as the (re)starting point (“seed” node) for the random walker, an imaginary particle that visits the network in a predefined number of steps, each consisting of one of the following moves:

i. randomly move to a neighboring node on the same layer;
ii. jump to a linked node of the same type on a different layer;
iii. transit to a different node type through the gene–drug network;
iv. return back to the seed node.

All possible transitions for each node of the $G_{MH}$ graph are simultaneously explored and recorded step-by-step in a probability distribution vector ${\overset{―}{p}}_{t}$. At each step, the random walker has a certain probability of returning to the seed node and a complementary probability of moving across the existing edges based on its previous position. The progression of the random walker’s traversal can be described as in [18]:

(1) $${\overset{―}{p}}_{t+1}^{T}=(1-r)H{\overset{―}{p}}_{t}^{T}+r{\overset{―}{p}}_{RS}^{T},$$

where the parameter r is the restart probability, ${\overset{―}{p}}_{t+1}$ and ${\overset{―}{p}}_{t}$ are the probability distribution vectors at the next step and at the current step, respectively, and H is a column-normalized transition matrix (see Supplementary Data). Furthermore, ${\overset{―}{p}}_{RS}$ represents the initial restart probability distribution vector, and is defined as:

$${\overset{―}{p}}_{RS}=\tau \cdot {\overset{―}{p}}_{0},$$

where ${\overset{―}{p}}_{0}$ is a binary vector in which non-zero elements represent the positions associated with the projections of the seed node in each layer, while the vector parameter τ measures the probability of restarting in the seed node of each layer. As the parameter r decreases, the random walker will step away from the seed node while diffusing across the MH network. Therefore, the choice of r affects the diffusion radius of the random walker from the seed. Moreover, the choice of the vector parameter τ is useful to prioritize layers during the restart process.

At the stationary state, each probability distribution vector ${\overset{―}{p}}_{s}$ represents the proximity between the associated seed node and all other nodes in each layer of the $G_{MH}$ network. This vector has a length equal to $\left(|Genes|\times L+|V_{DB}|\right)$. To generate a global similarity score, the geometric mean, arithmetic mean, or sum can be applied, producing a $\left(|Genes|+|V_{DB}|\right)$ proximity vector for each node. The resulting scaled $\left(|Genes|+|V_{DB}|\right)$ vectors ${\overset{―}{p}}_{ss}$, obtained from all RWRA iterations, are concatenated column-wise to create a RWR-MH similarity matrix where each column j indicates the multi-omics proximity between node j and all other nodes.

### 2.6 Model interpretability

The relative stationary probability distribution vectors ${\overset{―}{p}}_{s}$, generated during the RWRA, are used in MiDNE to compute a measure of the contribution of each omics layer to the proximity score between any pair of nodes. Given a pair of nodes $\left(node_{1},node_{2}\right)$, the L proximity values associated with $node_{2}$ in the proximity vector ${\overset{―}{p}}_{s1}$ of $node_{1}$ are used to define the relative omics contribution.

### 2.7 Embedding and dimensionality reduction

The embedding of the RWR-MH similarity matrix is performed using the MultiVERSE algorithm [28]. This step, while not mandatory, proves beneficial for de-noising the RWR-MH matrix, ensuring proper scaling and normalization are preserved. In addition, other dimensionality reduction functions – such as Principal Component Analysis (PCA), Uniform Manifold Approximation and Projection (UMAP) [29], and t-distributed Stochastic Neighbor Embedding (t-SNE) [30] – can be used to further reduce the dimensionality of the input (embedded) matrix.

### 2.8 Visualization

After the dimensionality reduction step, it is possible to analyze each nodes’ neighborhood in the generated multi-omics projection to evaluate gene–gene, gene–drug and drug–drug associations. In particular, the multi-omics projection of genes and drugs can be visualized by plotting the first two principal components of the PCA reduction matrix or by using the UMAP or t-SNE projections. The interactive visualizations are generated in MiDNE using the ggplot2 [31] and plotly [32] R packages.

### 2.9 Clustering analysis and enrichment analysis

Clustering of genes/drugs coded in the multi-omics projection, can be performed using one of the following clustering algorithms: i) k-means, ii) hierarchical clustering, or iii) Density-Based Spatial Clustering of Applications with Noise (DBSCAN). The functional annotation of obtained clusters is performed by over-representation analysis of the set of genes that are present in each cluster across all gene sets collections provided by the gprofiler R package [33].

### 2.10 Evaluation metrics

Functional annotations for gene nodes were retrieved from Gene Ontology (GO), KEGG, and Reactome databases. For each of the above annotations, a binary adjacency matrix encoding each gene co-occurrence with other genes in a functional term was built. For drug–gene relationships, the list of genes involved in each drug-related pathway was retrieved using DrugBank annotations linking drugs to pathways in the Small Molecule Pathway Database (SMPDB) [34]. A drug × gene binary matrix was then generated, where an entry equals 1 if a gene appears in at least one pathway associated with the corresponding drug. Gene- and drug-oriented co-participation profiles were then used as class labels for a silhouette analysis with respect to their euclidean distance in the corresponding embedding space or the length of the connecting shortest path in the Decagon PPI [26].

## 3 Results

### 3.1 MiDNE pipeline

**Fig. 1:**
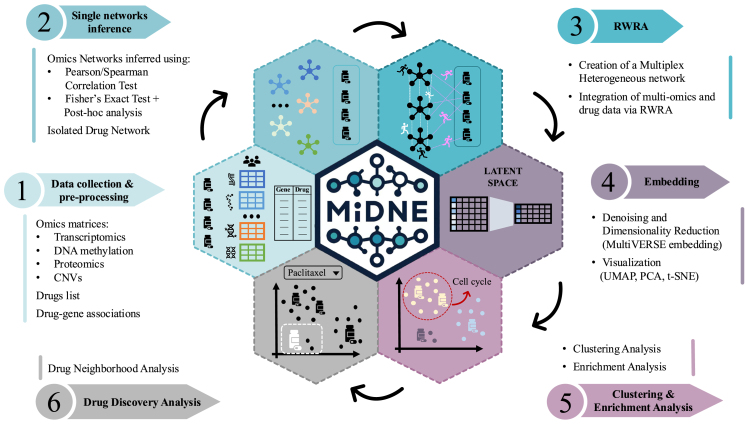
MiDNE pipeline for the integration and interpretation of multi-omics and drugs data.

The MiDNE integration pipeline is initialized with a list of one or more gene level omics matrices and a list of drug–gene associations. Omics matrices are passed to the tool as numeric matrices with genes placed on rows and samples on columns while gene–drug associations are provided as a two-column table respectively containing target gene and drug identifiers (Fig. 1:**1**). In the first step, a gene–gene network is inferred for each omics layer by applying a selected statistical association test to all gene pairs. Edges are established between gene pairs that meet a user-defined threshold on the test statistics, reflecting meaningful relationships specific to the omic modality. Depending on the nature of the omics layer, MiDNE supports different association measures: Pearson or Spearman correlation for quantitative data (e.g., gene expression), and Fisher’s exact test-based procedure for binary data (e.g., mutation or discretized methylation events) (Fig. 1:**2**, Table S.1). Single-omics networks are connected into a comprehensive network, by linking nodes associated with the same gene (or their direct neighbors) between different omics networks and adding a second set of nodes associated with drugs connected with the respective target genes as reported in the provided gene–drug association list. In the subsequent step, MiDNE computes multi-omics similarity profiles for all nodes by first applying the RWRA diffusion-based algorithm to the integrated network for each node (Fig. 1:**3**), followed by embedding the resulting similarity values into a low-dimensional matrix (Fig. 1:**4**). This matrix forms the basis for exploring multi-omics relationships.

Specifically, functional enrichment analysis can be performed on groups of genes (Fig. 1:**5**), manually selected from a two-dimensional visualization of the embedding or identified by clustering algorithms applied to the embedded proximity matrix, to functionally annotate multi-omics gene–gene associations. Additionally, gene–drug and drug–drug interactions can be explored by examining the multi-omics neighborhood of a drug of interest (Fig. 1:**6**). Finally, MiDNE supports the interpretability of multi-omics associations by providing single-omics proximity weights for user-selected node pairs.

### 3.2 MiDNE R package

MiDNE is available as an R package providing a comprehensive suite of functions, including: (i) importing, normalizing, and filtering omics and pharmacological data; (ii) building gene networks; (iii) integrating multiple omics networks; (iv) performing dimensionality reduction; (v) visualizing results; (vi) annotating data with clustering information, enrichment terms, or user-provided annotation tables; and (vii) assessing the contribution of individual omics layers to multi-omics associations. The MiDNE package also offers an interactive graphical user interface, implemented using the Shiny R package [35] (Figures S.1–8).

### 3.3 Case study: BRCA and GBM

MiDNE was benchmarked for modeling multi-omics interactions on two TCGA and CPTAC datasets, using FDA-approved drugs to identify cancer-specific, targetable genes. Specifically, we selected and processed four BRCA and three GBM datasets, including DNA methylation, transcriptomic, CNV and proteome data (this latter for BRCA only) (Table 1 and S.2). In the first step, for each cancer type, the omics matrices were provided as input to MiDNE, which inferred distinct gene networks (Table 1 and S.2), as described in Section 2.2. The resulting co-expression and protein co-abundance networks were filtered to control network density – measured by the number of edges – while retaining as many features as possible, using an absolute correlation threshold ≥0.5 and an adjusted p-value <0.05. Similarly, the co-methylation, co-amplification, and co-deletion networks were filtered to exclude gene pairs with low or non-significant co-occurrence frequencies.

**Table 1:** Description of the BRCA multi-omics datasets utilized in this study and the corresponding inferred networks.

Omics name	Dimensions (genes × samples)	Inference method	Network type	N${\;}^{\circ }$ nodes (genes)	N${\;}^{\circ }$ edges
Transcriptomics	20,155 × 1093	Pearson Correlation Test	co-expression	15,317	1,678,694
Proteomics	9733 × 105	Spearman Correlation Test	co-abundance	7430	2,008,006
DNA Methylation	20,106 × 783	Fisher’s exact test	co-methylation	3870	612,888
CNV	24,776 × 1080	Fisher’s exact test	co-amplification	3446	1,348,893
			co-deletion	3393	761,632

Subsequently, the obtained omics networks were assembled into an unweighted multiplex omics network by linking nodes with their own projections across different layers. For BRCA, the latter consists of 19,929 nodes in each of the five layers, 6,410,113 total intra-layer edges, and 199,290 total inter-layer edges, while for GBM it consists of 19,592 nodes in each of the four layers, 5,007,568 intra-layer edges, and 117,552 inter-layer edges. For each cancer type, the gene–drug network in MiDNE was derived as the subset of known gene–drug associations involving genes present in the corresponding multiplex omics network. The resulting gene–drug network comprises 698 genes and 1450 drugs for BRCA, and 736 genes and 1455 drugs for GBM. Then, a MH network has been generated for each cancer by linking drug nodes with corresponding target gene nodes in the multiplex omics network. The RWRA was then applied to each cancer-specific MH network to integrate gene and drug data. The resulting node similarity matrices were finally embedded in a 1000-factor latent space using the MultiVERSE procedure (Section 2.7). The UMAP projections of the embedded similarity matrix for BRCA and GBM are shown in Figures S.9 and S.10, respectively. Execution time and memory usage were profiled for each step of the MiDNE pipeline in both cancer types and are reported in Figure S.11.

### Evaluation of MiDNE and baselines

**Fig. 2:**
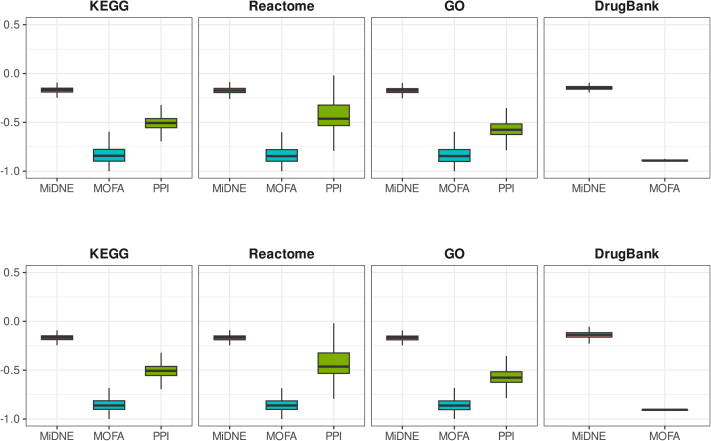
Silhouette score distributions of genes and drugs comparing MiDNE and MOFA embeddings for BRCA (**A**) and GBM (**B**) multi-omics datasets, along with results inferred from a generic PPI network. KEGG, Reactome, GO, and DrugBank were used as reference databases. For MiDNE, the best performance was obtained with 1000 latent factors, whereas for MOFA an optimal performance was achieved with 64 factors.

We compared the performance of MiDNE in recovering biologically functional associations against MOFA [4], a non-network-based method for multi-omics data integration. We also compared the ability of MiDNE to capture condition-specific associations with the relationships derived from the generic PPI network of the Decagon project [26]. To assess the ability of the MiDNE embedding to capture functional associations between nodes and to compare its performance with other integration methods, we developed a validation metric based on silhouette analysis of genes co-occurring in functional terms across multiple biological databases (KEGG, Reactome, and GO), or within drug-associated pathways (DrugBank and SMPDB), relative to their distances in the embedding space or the lengths of their shortest connecting paths in the Decagon PPI network (Supplementary Data). The euclidean distance was computed on MiDNE 1000-factor matrix, and MOFA 64-factor matrix respectively. MiDNE consistently achieved higher scores compared to other baseline methods in all the considered biological databases for both BRCA (Fig. 2**A** and S.12**A**) and GBM (Fig. 2**B** and S.12**B**) datasets.

## 4 Discussion

In this manuscript, we present MiDNE, a bioinformatics tool that, starting from multi-omics data and drug information, models biological and pharmacological entities into a unified space. MiDNE is designed not only to uncover drug–gene associations, but also to model condition-specific gene–gene and drug–drug interactions, providing a more comprehensive view of the dynamics underlying molecular interactions. Moreover, MiDNE implements functionalities that are not jointly available in other computational frameworks. First, it provides network inference methods specifically tailored to the intrinsic properties of different biological layers. In particular, MiDNE generates omics-specific networks from expression, proteomic, CNV, and DNA methylation data. Unlike other methods, such as AMEND [15] and DrugComboExplorer [16], which use DNA methylation and CNV data solely for feature selection and prioritization, MiDNE models gene associations based on the co-occurrence frequency of CNV and methylation events across the analyzed samples. Moreover, by representing biological systems as a multiplex network, MiDNE can accommodate an arbitrary number of input omics matrices, handle missing observations across omics layers, and integrate additional data types, such as drugs. An additional strength of the tool is the inclusion of a trace-back mechanism, which enables the analysis of the contributions of individual omics layers to the discovered associations.

The ability of MiDNE to capture functional associations among genes and drugs was evaluated against a state-of-the-art multi-omics integration method, MOFA, using two multi-omics datasets. MiDNE achieved higher silhouette scores than MOFA across different reference databases (Fig. 2) and embedding sizes (Figure S.12). In particular, we observed a strong dependency between the number of latent variables and the performance of both methods: larger embedding sizes were generally associated with higher silhouette scores. However, this trend did not hold for the MOFA drug silhouette score, which reached its highest performance with 16 latent factors. The higher performance of MiDNE compared to MOFA may be attributed to differences in their integration models. While MOFA operates directly on the original omics matrices, MiDNE leverages network transformation to reduce the intrinsic noise of the data, thereby shaping functional associations that are further refined during the embedding step.

Moreover, the multi-molecular associations captured by MiDNE when modeling condition-specific omics datasets were evaluated against those described in a generic PPI network by calculating gene silhouette scores as done previously (Fig. 2). MiDNE scores were higher than those of the PPI network across all three considered functional databases, likely because MiDNE captures deeper functional associations arising from interactions across multiple molecular domains.

From a computational perspective, by using the BRCA-MH network as a test case, we found that MiDNE’s results are highly stable across variations in the RWRA parameters, particularly the inter-layer transition probability (δ) and the gene–drug transition probability (λ) (Figure S.13**A,C**). However, slight differences were observed when prioritizing a specific layer during the restart process compared with using equal restart probabilities across layers (Figure S.13**B,D**). In this case, the most pronounced discrepancy occurs when prioritizing the co-expression layer, followed by the protein co-abundance, co-methylation, and co-CNV networks, which is consistent with the varying relevance of molecular domains in biological information flow. These differences can be beneficial in scenarios requiring layer-oriented integration. Finally, variation in the restart parameter (r) has a more pronounced effect on the RWR similarity matrix (Figure S.13**A,C**), as it determines the size of the node’s neighborhood explored during the diffusion process and, consequently, the resulting probability profile.

## 5 Conclusion

This work aimed to provide the scientific community with a bioinformatics tool for multi-molecular investigation of complex systems by generating disease-specific gene and drug multi-omics models. From a computational perspective, MiDNE demonstrated broad applicability as an unsupervised multi-view integration tool. While it was applied primarily to bulk tumor datasets in this work, MiDNE could be extended to specific tumor subtypes and single-cell data, allowing the derivation of patient-specific integrated gene models. These features highlight MiDNE’s potential to support more precise and comprehensive analyses of complex biological systems. MiDNE is available as an R package along with a user-friendly Shiny app at https://github.com/BioinfoUninaScala/MiDNE.

## CRediT authorship contribution statement

**Aurora Brandi:** Writing – original draft, Writing – review & editing, Software, Formal analysis, Data curation, Visualization. **Barbara Majello:** Writing- Reviewing and Editing, Supervision, Funding acquisition. **Ines Simeone:** Writing – original draft, Writing- Reviewing and Editing. **Massimiliano Romano:** Software. **Michele Ceccarelli:** Writing – original draft, Conceptualization, Resources. **Giovanni Scala:** Writing – review & editing, Supervision, Conceptualization, Resources.

## Declaration of competing interest

The authors declare that they have no known competing financial interests or personal relationships that could have appeared to influence the work reported in this paper.

## Data Availability

The datasets generated and analyzed in this study, along with the corresponding code, are available at https://github.com/BioinfoUninaScala/MiDNE.
