# Supplementary material for: MiDNE a tool for Multi-omics genes and drugs interactions discovery
Source: Comput Struct Biotechnol J. 2025 Oct 15;27:4469–75. doi: 10.1016/j.csbj.2025.10.022 (PMC12593681; doi:10.1016/j.csbj.2025.10.022)
Supplement: Multimedia Component 1 [file mmc1.pdf]

Supplementary Data

MiDNE a tool for multi-omics genes and drugs interactions discovery

0.1. Omics networks inference

Definition. Starting from N omics matrices, MiDNE generates a collection of L undirected gene networks denoted $G_\alpha = (V_\alpha, E_\alpha)$, with $\alpha = \{1, \ldots, L\}$, where each α is associated with a particular biological layer of information. MiDNE infers the omics networks using different statistical metrics that are chosen based on the omics considered (Table S.1).

Table S.1: Statistical methods utilized by MiDNE to infer the weighted omics networks based on the input omics type.

**Omics type**	**Inference method** sig_α	**Network type**	**Weight type** $stat_\alpha$
Transcriptomics	Pearson Correlation Test	co-expression	Pearson Correlation Coefficient
Proteomics	Spearman Correlation Test	co-abundance	Spearman Correlation Coefficient
DNA Methylation		co-methylation	
CNV	Fisher's exact test	co-amplification	$\log\left(\frac{expected}{observed}\right)$
		co-deletion	

Binarization of copy number alterations. The CNV matrix is used to derive two binary matrices that separately represent amplification (positive values) and deletion (negative values) events. In the amplification matrix, a value of 1 codes for an amplification event, while a value of 0 indicates the absence of amplifications. In the deletion matrix, a value of 1 codes for a deletion event, while a value of 0 indicates the absence of deletions.

Fisher's exact test for pairs of genes. The contingency matrix for a gene pair ij (with $i \neq j$) has rows representing states 0 and 1 for gene i and columns representing states 0 and 1 for gene j, where 0 and 1 indicate, respectively, the absence and presence of the associated molecular event (methylation, deletion or amplification). Hence, the cell with coordinates (0,0) indicates the co-absence of the event, the cells with coordinates (0,1) and (1,0) correspond to mutually exclusive events, and the cell with coordinates (1,1) represents the co-occurrence of the event. A post hoc analysis is performed to select

gene pairs for which the null hypothesis has been rejected due to the presence of a significant imbalance in favor of the (1,1) cell over the others.

0.2. Random Walk with Restart (RWR)

RWR on Multiplex Networks. The Random Walk with Restart (RWR) algorithm on a multiplex network is defined by the iterative equation:

$$\overline{p}_{t+1}^{T} = (1-r)A\,\overline{p}_t^{T} + r\,\overline{p}_{RS}^{T}, \tag{S.1}$$

where $\overline{p}_t$ is the probability distribution vector of the random walker at time step t, A is the column-normalized adjacency matrix of the multiplex network, $r \in [0,1]$ is the restart probability, and $\overline{p}_{RS}$ is the initial restart probability distribution vector. Specifically, the latter is defined as $\overline{p}_{RS} = \tau \cdot \overline{p}_0$, where $\overline{p}_0$ is a binary vector in which the only non-zero elements are the positions associated to the projections of the seed node in each layer, while the vector parameter τ measures the restart probability in the seed of each layer.

In a multiplex network composed of L layers, the matrix A takes the following block structure:

$$A = \begin{bmatrix} (1-\delta)A^{[1]} & \frac{\delta}{L-1}T^{1,2}c^{1,2} & \cdots & \frac{\delta}{L-1}T^{1,L}c^{1,L} \\ \frac{\delta}{L-1}T^{2,1}c^{2,1} & (1-\delta)A^{[2]} & \cdots & \frac{\delta}{L-1}T^{2,L}c^{2,L} \\ \vdots & \vdots & \ddots & \vdots \\ \frac{\delta}{L-1}T^{L,1}c^{L,1} & \frac{\delta}{L-1}T^{L,2}c^{L,2} & \cdots & (1-\delta)A^{[L]} \end{bmatrix}, \tag{S.2}$$

where A^{α} is the intra-layer column-normalized adjacency matrix of layer $\alpha = [1,\ldots,L]$, the parameter $\delta \in [0,1]$ controls the inter-layer transition probability, T is a binary matrix indicating whether transitions are allowed from layer α to layer β, and c is a probability matrix defining the relative transition probability between layer α and layer β.

RWR on Multiplex Heterogeneous Networks. We extend the RWR framework to a heterogeneous multiplex network G_{MH} integrating a multiplex gene network and a drug network through bipartite interactions. The Equation S.1 is thus reformulated as:

$$\overline{p}_{t+1}^{T} = (1-r)H\,\overline{p}_t^{T} + r\,\overline{p}_{RS}^{T}, \tag{S.3}$$

where H is the global transition matrix of the multiplex heterogeneous network, defined as:

$$H = \begin{bmatrix} H_{G-G} & H_{G-D} \\ H_{D-G} & H_{D-D} \end{bmatrix}, \tag{S.4}$$

where H_{G-G} represents transition probabilities within the multiplex gene network, H_{D-D} accounts for transition probabilities within the drug network, and H_{G-D} and H_{D-G} model the transitions across the bipartite gene–drug connections. In particular, the sub-matrix H_{G-G} is derived from the multiplex transition matrix A defined in Equation S.2. However, when a gene node is connected to one or more drug nodes, the transition probabilities within the gene multiplex G_M are rescaled by a factor $(1 - \lambda)$, with λ representing the global transition probability across the bipartite network. Conversely, the sub-matrices H_{G-D} and H_{D-G} explicitly depend on λ, as they govern the transition probabilities across the bipartite gene–drug network G_{DB}.

Parameters setting. The Random Walk with Restart algorithm (RWRA) has been applied to two multi-omics heterogeneous multiplex networks (i.e., breast invasive carcinoma and glioblastoma multiforme). The restart probability was set to $r = 0.7$, and the restart vector $\tau = \left(\frac{1}{L}\right)_{i=1}^{L}$ was used to assign uniform restart probabilities across the L omics layers. The inter-layer transition probability was set to $\delta = 0.5$, and the transition layer matrix c modeled uniform transition probabilities between the omics layers. Specifically, in the matrix c, all off-diagonal elements are set to $\frac{1}{(L-1)}$, while the on-diagonal elements are set to zero, ensuring that transitions can only occur between different omics layers with equal probability. Finally, the gene–drug network transition probability was set to $\lambda = 0.5$.

Evaluation of different RWRA parameters. To assess the impact of RWRA parameter variations on MiDNE results, we calculated pairwise Normalized Mutual Information (NMI) scores between the RWRA proximity matrix obtained with the reference parameters (as described in the *Parameter settings* section of the Supplementary Materials) and those derived using alternative parameter configurations. The evaluation was performed using BRCA multi-omics data and drug-target associations from the DrugBank database as test datasets. We explored the effects of using different values for the restart parameter r, the inter-gene-layer transition probability δ and the gene–drug transition probability λ, all ranging from 0 to 1 (extreme values excluded). Specifically, we varied one parameter at a time from 0.1 to 0.9 in increments of 0.2, while keeping the other two fixed at 0.5 to isolate its effect, and recomputed the corresponding node proximity matrices (Figure S.13A-C). We observed consistently higher NMI values between the reference proximity vectors and those inferred by varying δ or λ, while a moderate drop in NMI

was observed by decreasing the value of the parameter r. This trend can be explained by the relationship between the restart probability r and the size of the neighborhood explored by the random walker when it restarts in a certain seed: in fact, as r decreases, the random walker explores an increasingly larger neighborhood, thus affecting the resultant stationary probability distribution vector. We also investigated the impact of layer prioritization during the restart process: we performed the RWR step 5 times, each time assigning a value of 0.6 to the prioritized layer in the restart probability vector τ, and a value of 0.1 to the others (Figure S.13B-D). We found that prioritizing the expression layer yielded a lower NMI than prioritizing other layers. The reduced stability may reflect the stronger influence of the expression layer's topology in shaping node neighborhoods.

Computational Efficiency. In order to test the performance of MiDNE in terms of speed and memory usage, we applied its framework to BRCA and GBM multi-omics datasets using an increasing number of processors (i.e., 1, 10, 100) for each step. Specifically, we separately profiled the runtime and memory usage for the network inference step, the integration step through RWRA, and the embedding step using the MultiVERSE method [28]. The results are shown in Figure S.11. All computations were performed on a computing server equipped with two AMD EPYC 7713 64-core processors and 1 TB of memory.

Evaluation of MiDNE and MOFA. We compared the performance of MiDNE with MOFA [4], a non-network-based method for multi-omics data integration. MOFA utilizes Factor Analysis to generate a low-dimensional representation of the data that can be directly compared with the embedding inferred by MiDNE. We applied MOFA to the BRCA and GBM datasets by providing it with cancer-specific omics matrices and an additional binary matrix that models gene–drug associations. To enable the application of MOFA, we manually inserted columns of missing values to ensure that all input matrices shared the same set of features. We then calculated a silhouette-based score using the Euclidean distance matrix of both embeddings and gene sets from several databases (i.e., KEGG, Reactome, GO, DrugBank) to assess the ability of the two methods to capture functional associations between genes and drugs (Methods). MiDNE consistently achieved higher silhouette scores compared to MOFA in all biological databases for both cancer types and for different numbers of latent factors (10, 16, 32, and 64) (Figure S.12).

At 64 latent factors, MOFA showed computational limitations and warnings indicating longer processing times.

Evaluation of MiDNE and PPI. We compared the results obtained by applying the MiDNE framework to BRCA and GBM datasets with that derived from a generic protein–protein interaction (PPI) network. This analysis was designed to evaluate the ability of MiDNE to capture context-specific gene functional associations. Specifically, we collected a PPI network from the Decagon project [26], which includes 715,612 physical and functional interactions between 19,081 proteins. A gene–gene distance matrix was generated by computing the shortest path between all pairs of genes in the PPI, and then used to calculate the silhouette score distribution with respect to the KEGG, Reactome and GO datasets. MiDNE applied to both cancer-specific multi-omics datasets achieved higher silhouette scores compared to a generic PPI according to all reference datasets.

Table S.2: Description of the GBM multi-omics datasets utilized in this study and the corresponding inferred networks.

**Omics name**	**Dimensions** (genes × samples)	**Inference method**	**Network type**	**N° nodes** (genes)	**N° edges**
Transcriptomics	19,660 × 153	Pearson Correlation Test	co-expression	17,491	2,920,225
DNA Methylation	20,114 × 140	Fisher's exact test	co-methylation	3,927	406,697
CNV	24,776 × 577		co-amplification	2,689	1,149,045
			co-deletion	1,555	531,601

Supplementary Figures

MiDNE a tool for multi-omics genes and drugs interactions discovery

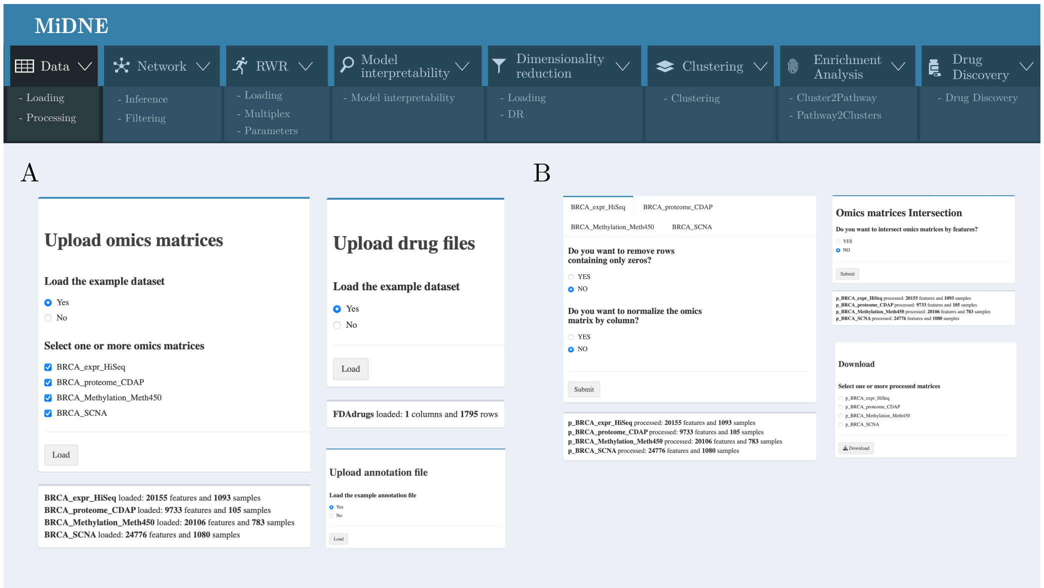

Figure S.1: First panel of the MiDNE shiny app, consisting of the "Loading" (A) and "Processing" (B) sub-panels. (**A**) Users can upload omics matrices (genes as rows and samples as columns), a list of drug identifiers, and an annotation file reporting node labels, node types, and additional information used to color nodes in subsequent steps. Alternatively, example datasets can be uploaded, including breast invasive carcinoma data from the TCGA and CPTAC projects, a list of FDA-approved drugs from DrugBank, and the corresponding annotation file. (**B**) Users can process the uploaded omics matrices by removing zero rows (if any), scaling data by columns, and selecting only the common features across all matrices. The processed matrices can then be downloaded from the corresponding panel.

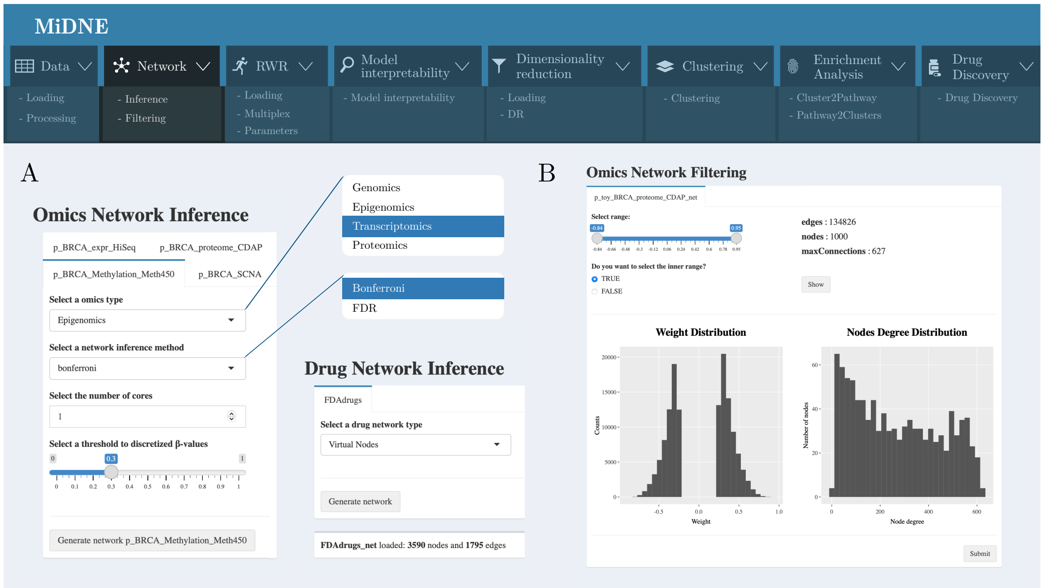

Figure S.2: Second panel of the MiDNE shiny app, consisting of the "Inference" (A) and "Filtering" (B) sub-panels. (**A**) For each uploaded and processed matrix, users can construct omics networks according to the selected input omics type (i.d., correlation test for transcriptomics and proteomics data, and Fisher's exact test for genomics, epigenomics data), the chosen correction method for the multiple testing (Bonferroni or FDR) and the number of CPUs to run in parallel. Additionally, for epigenomics data, user can select a threshold on β values to binarize the data. If a drug list is provided, an isolated drug network is also constructed. (**B**) The inferred omics networks can then be filtered individually based on the edge weights: for correlation-based networks, user can retain edges within a specified lower and upper range, while for co-occurrence networks, only edges with values above a given threshold are retained. The figure shows an example of a protein co-abundance network. After selecting the threshold, clicking the "Show" button displays the updated topological properties of the network, including the number of nodes and edges, node degree distribution, and weight distribution. The final filtered network is saved and passed to the subsequent step by clicking the "Submit" button.

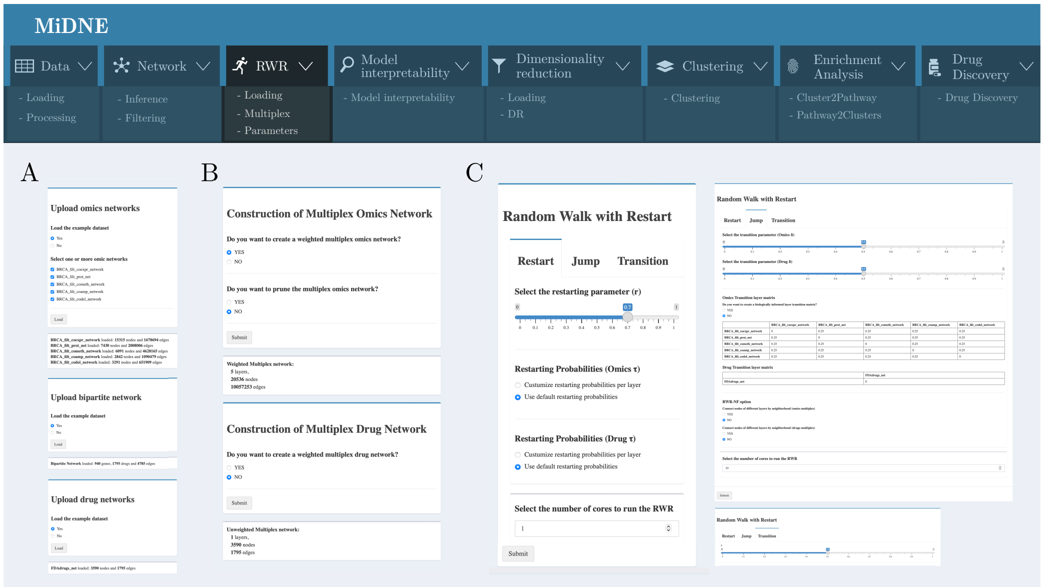

Figure S.3: Third panel of the MiDNE shiny app, consisting of the "Loading" (A), "Multiplex" (B) and "Parameters" (C) sub-panels. (**A**) Users can initiate the analysis from this panel by uploading gene and drug networks. Alternatively, example datasets can be uploaded, including five breast invasive carcinoma networks inferred from TCGA and CPTAC data, an isolated network of FDA-approved drugs, and the known gene-drug associations from DrugBank. Whether the networks are inferred in the second panel or uploaded directly here, it is necessary to also provide a gene–drug network to define the bipartite associations within the multiplex heterogeneous network. (**B**) In this panel, users can build the multiplex omics network, and — if drug network is available — the multiplex/monoplex drug network as well. Users can choose whether to retain the edge weights of individual layers and optionally apply a global filtering step to all layers simultaneously. (**C**) Here, users can set the RWRA parameters, which are organized in three main categories: i) restart parameters (Restart tab), including the global restart parameter r, and the layer-specific restart vector parameter τ for each multiplex network (if a network is a monoplex, $\tau = 1$); ii) the inter-layer transition parameters (Jump tab), including the global inter-layer parameter δ within each multiplex network (if a network is a monoplex, δ is not considered), the inter-layer transition probability matrix used to tune the jump probability between pairs of layers, and the selected modality for connecting layers within a multiplex network (see *Methods* for details); iii) inter-multiplex parameters (Transition tab), including the gene-drug transition parameter λ. Finally, users can specify the number of CPUs to be used for parallel computation.

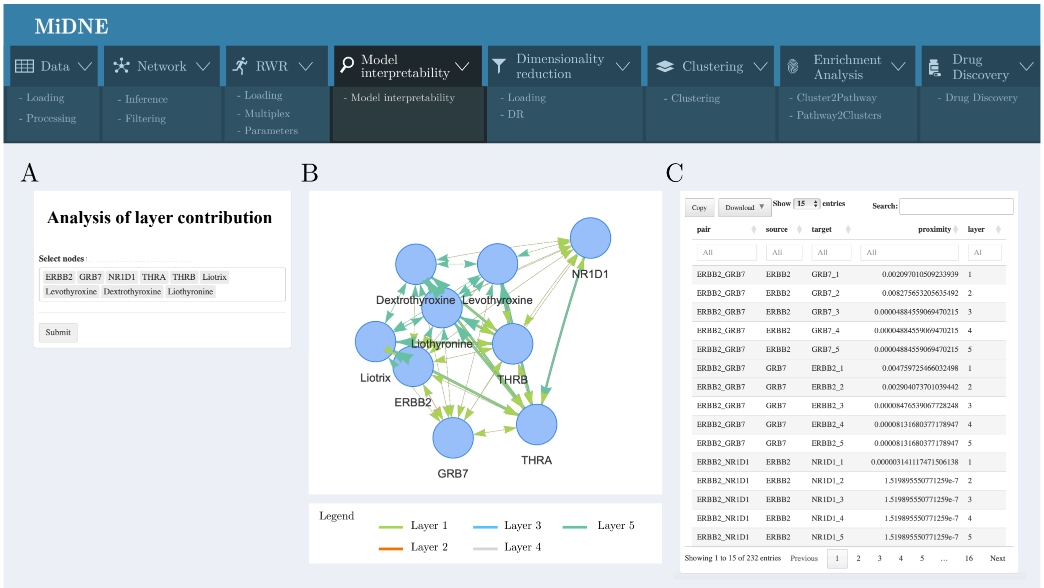

Figure S.4: Fourth panel of the MiDNE shiny app. (**A**) Here, users can explore the contribution of each layer to the node associations detected during the diffusion process modeled by the RWRA (see Fig. S.3) by selecting at least two nodes from the dedicated panel. (**B**) Each selected node is displayed as a node within the network, where the color and thickness of the edges represent the contribution of a given layer to their associations in terms of the random walk visiting probability. (**C**) Table reporting the contribution of each layer to every possible pair of selected nodes.

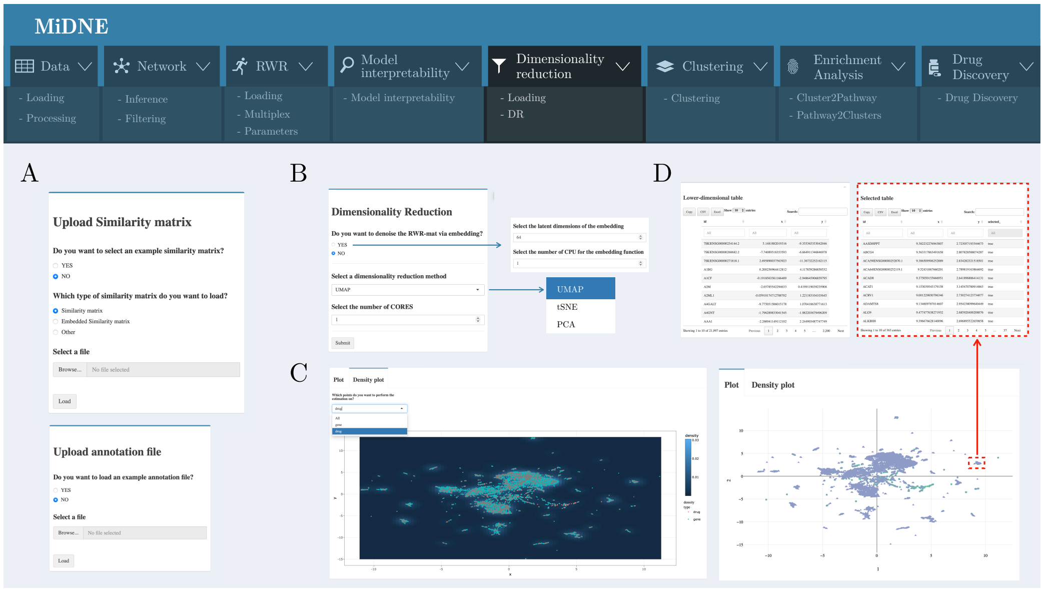

Figure S.5: Fifth panel of the MiDNE shiny app, consisting of the "Loading" (A) and "Dimensionality Reduction" (DR) (B-D) sub-panels. (**A**) Users can initiate the analysis from this panel by uploading either a RWRA similarity matrix, or an embedded RWRA similarity matrix, or other file types such as a 2D representation of the RWRA similarity matrix. In addition, user can update an annotation file containing node identifiers and types. Alternatively, example datasets can be uploaded, including the RWRA similarity matrix modeled for breast invasive carcinoma datasets and FDA-approved drugs from DrugBank. (**B**) If an RWRA similarity matrix is uploaded or created in the third panel (Fig. S.3), users can denoise it using the MultiVERSE procedure by selecting the number of latent variables and the number of CPUs. If an embedded similarity matrix is uploaded or created in the previous step, users can choose among UMAP, PCA, or tSNE methods to further reduce dimensionality for visualization purposes. (**C**) The figure shows two interactive UMAP visualizations for the example dataset: a density plot to examine how genes, drugs, or both aggregate in the projection, and a scatter plot that allows users to select groups of nodes to be displayed in a dedicated table shown in (D). (**D**) UMAP tables corresponding to the full projection shown in (C) and to the selected node groups obtained through the brush selection function in plotly.

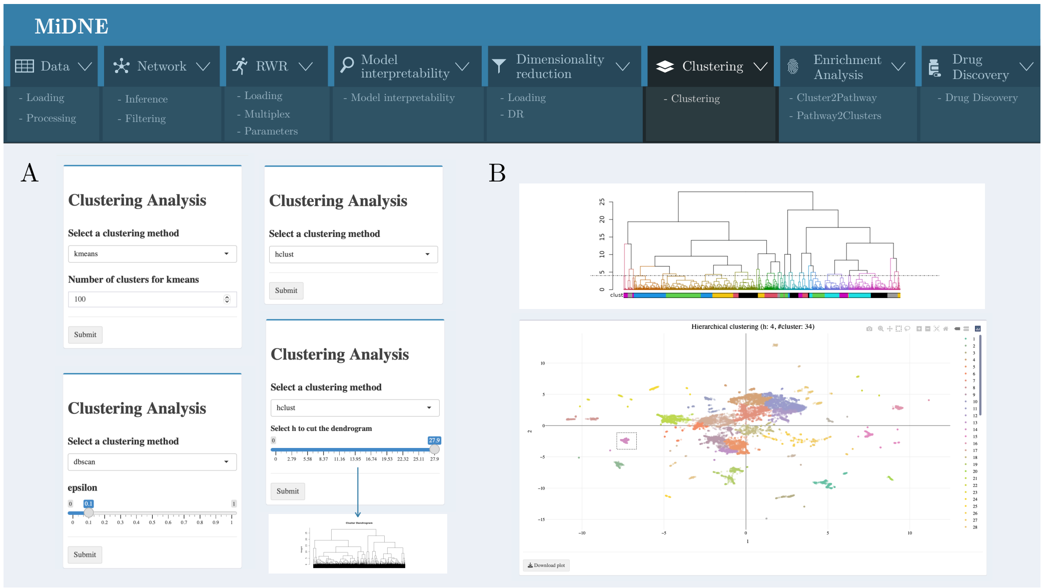

Figure S.6: Sixth panel of the MiDNE shiny app. (**A**) Users can choose among three clustering algorithms, including k-means, DBSCAN and hierarchical clustering (hclust), or manually select a group of nodes directly from the 2D projection. When using k-means or DBSCAN, users must specify the desired number of clusters and the parameter ϵ, respectively. When using hclust, a hierarchical tree is first constructed, and users can then select the cutting height to define the clusters. (**B**) The figure shows a UMAP visualization for the example dataset of breast invasive carcinoma integrated with FDA-approved drugs, where nodes are colored according to the cluster membership obtained via hierarchical clustering (bottom). The dendrogram is cut at a height of 4 to define the clusters (top).

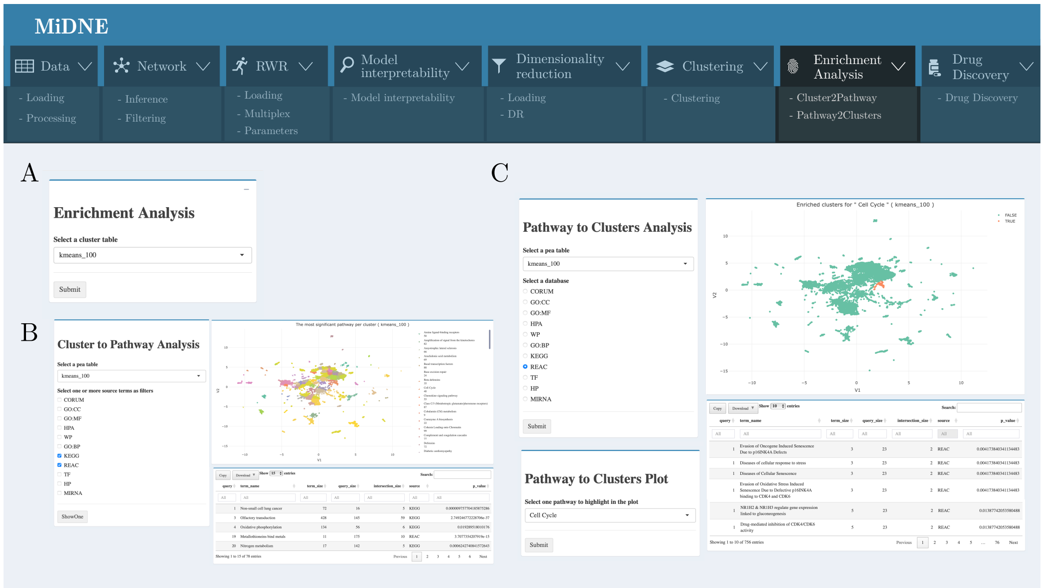

Figure S.7: Seventh panel of the MiDNE shiny app, consisting of the "Cluster2Pathway" (A-B) and "Pathway2Cluster" (C) sub-panels. (**A**) Users can select one of the cluster tables generated in the previous panel (Fig. S.6) to perform enrichment analysis using all databases available in gprofiler2 R package. (**B**) Users can then select one of the enriched cluster tables generated in (A), choose one or more datasets to filter it, and perform the "Cluster2Pathway" analysis. This analysis assigns to each cluster the most significantly associated term based on the adjusted p-value from the gprofiler2 analysis. In the top right corner, nodes in the 2D projection are colored according to the most significant term enriched for their corresponding cluster. In the bottom right corner, a table displays the one-to-one associations between clusters and enriched terms. (**C**) Users can select one of the enriched cluster tables generated in (A), choose a single dataset to filter it, and then perform the "Pathway2Cluster" analysis. In this panel, users can select any detected term from a specific dataset (bottom left corner) and highlight in the 2D visualization (top right corner) all clusters enriched for that term. In the bottom right corner, a table displays the multiple associations between clusters and dataset-specific terms.

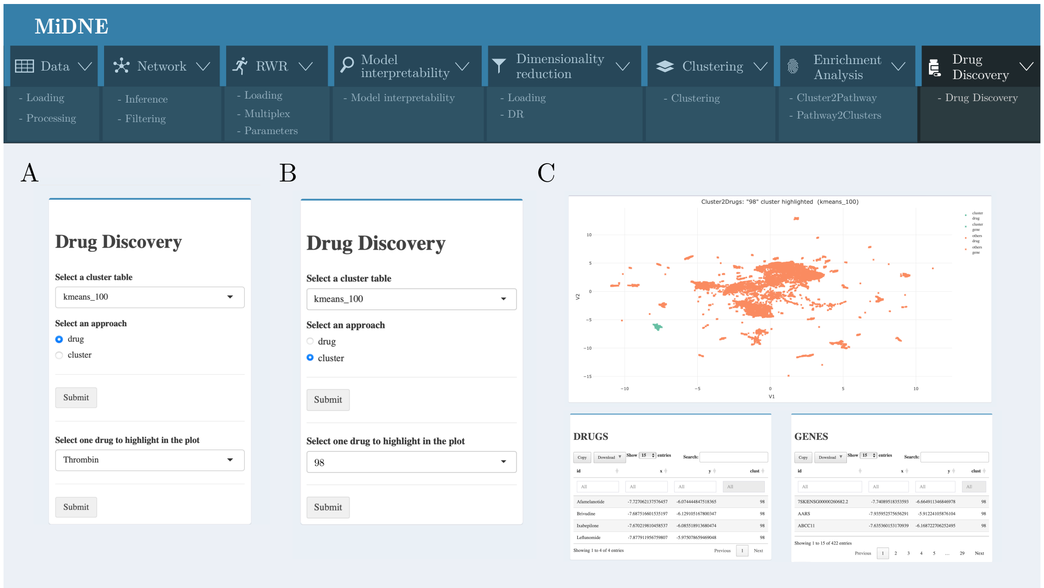

Figure S.8: Eighth panel of MiDNE shiny app. Users can select one of the cluster tables generated in the previous panel (see Fig. S.6) to associate drug nodes with their corresponding cluster memberships (A–B). (A) Users can select a specific drug of interest. (B) Alternatively, users can select a cluster of interest containing at least one drug. (C) The corresponding group of nodes is then highlighted in the 2D projection. The tables below the plot separately report the drug and gene nodes belonging to either the selected cluster or the cluster associated with the selected drug.

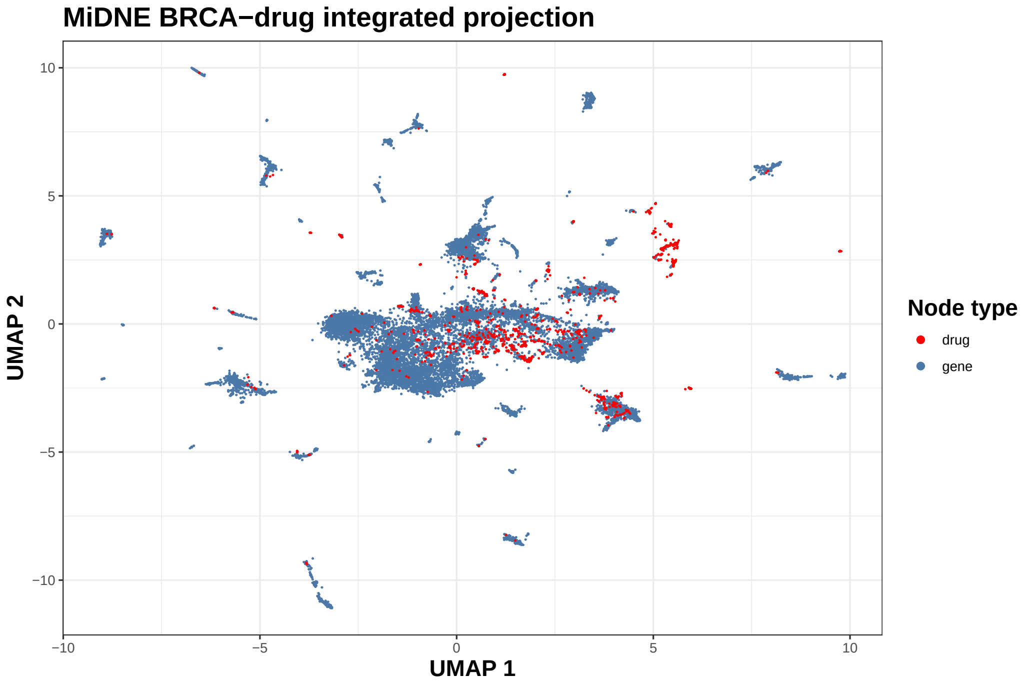

Figure S.9: UMAP projection of the 1000-factor embedded similarity matrix for 19, 929 BRCA genes (blue points) and 1, 450 drugs (red points).

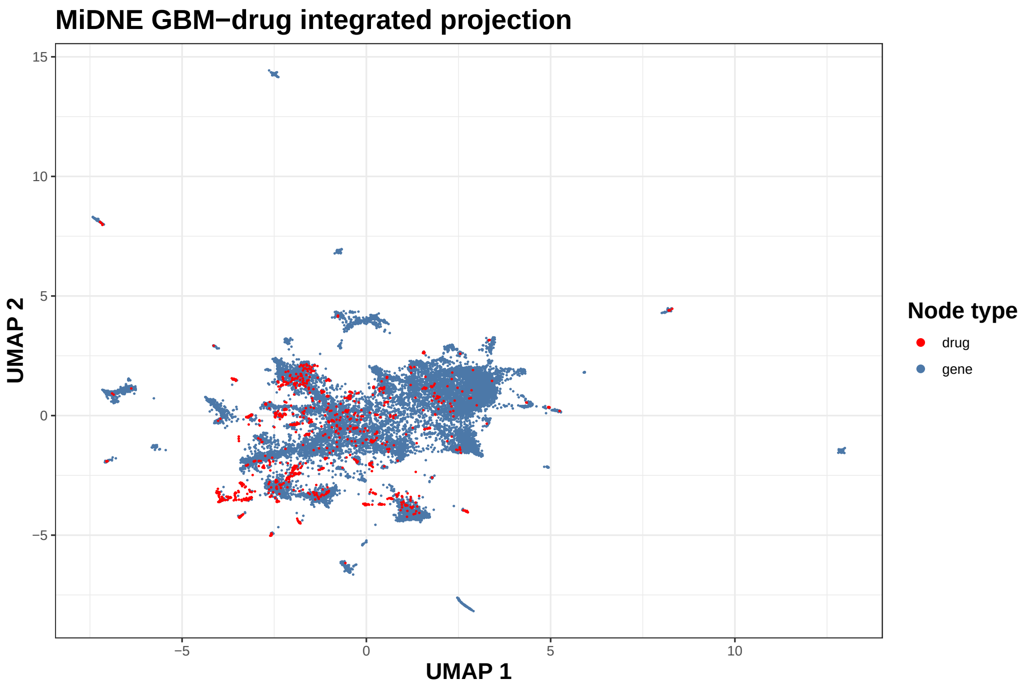

Figure S.10: UMAP projection of the 1000-factor embedded similarity matrix for $19,592$ GBM genes (blue points) and 1,455 drugs (red points).

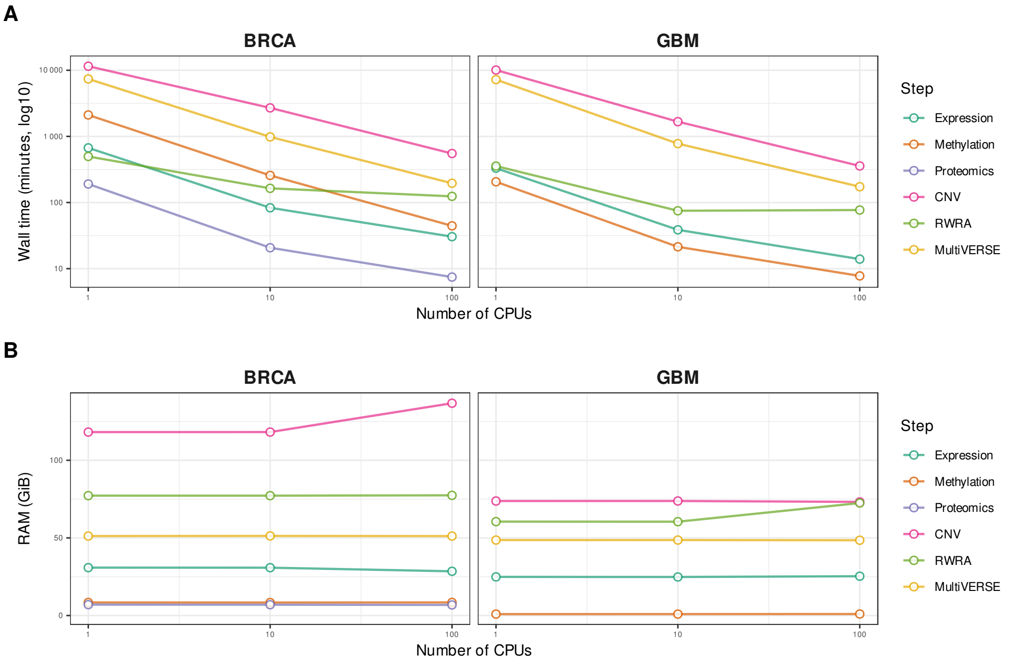

Figure S.11: Performance of MiDNE in terms of time (**A**) and memory usage (**B**) for single omics network construction steps and embedding execution steps (RWRA and MultiVERSE). The profiling analysis was performed on BRCA and GBM multi-omics datasets, using an increasing number of CPUs.

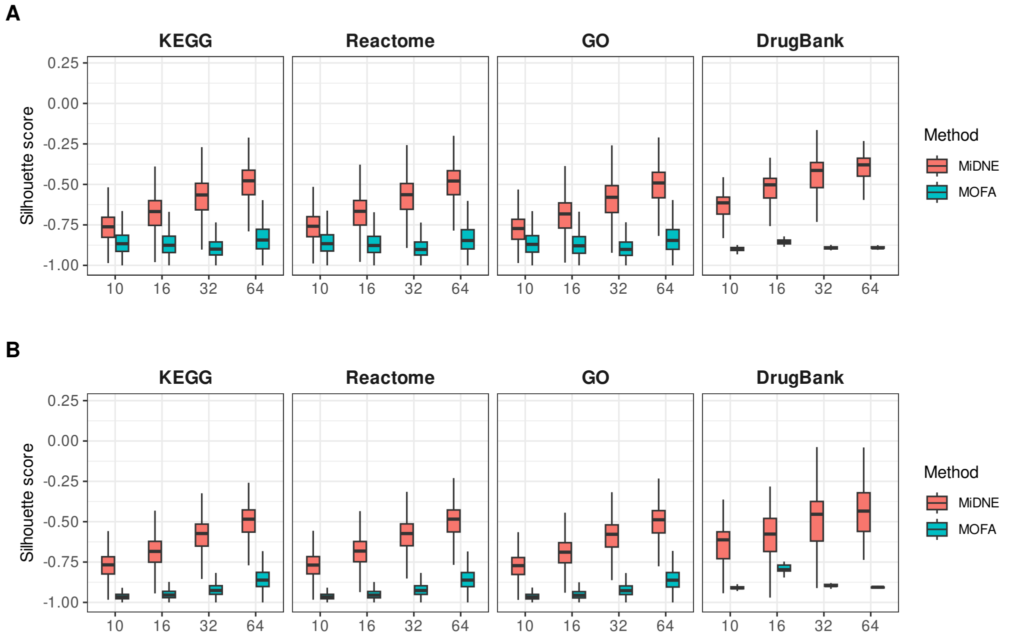

Figure S.12: Silhouette score gene/drug distributions comparing MiDNE embeddings and MOFA latent factors for BRCA (**A**) and GBM (**B**) multi-omics data. Gene silhouette scores were calculated using pairwise distances among genes co-occurring in any KEGG, Reactome, or GO gene sets, respectively, whereas drug silhouette scores were computed based on the distances among genes co-occurring in any of the corresponding drug pathway gene sets as reported in SMPDB. For each cancer type and reference dataset, the silhouette score is reported for different sizes of MiDNE and MOFA embeddings: 10, 16, 32 and 64.

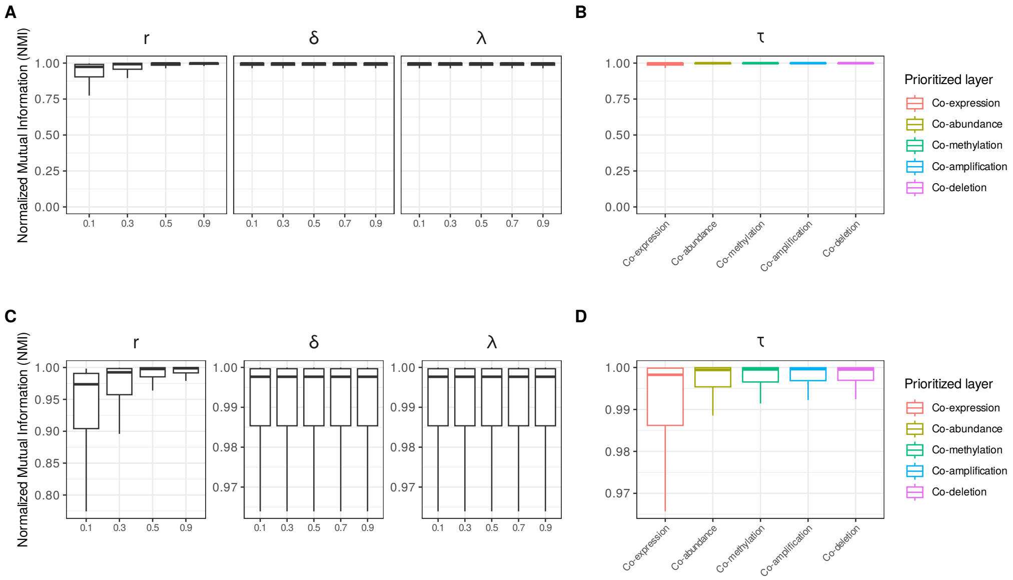

Figure S.13: Stability of MiDNE embeddings under parameter variation, evaluated using Normalized Mutual Information (NMI). (**A**) Distribution of NMI values comparing the RWRA proximity matrix computed with the reference parameter set (r=0.7, δ=λ=0.5) to those obtained by varying r, δ, or λ (0.1–0.9; remaining parameters fixed at 0.5). (**B**) Distribution of NMI values comparing the RWRA proximity matrix computed with the reference parameter set (tau=0.2 0.2 0.2 0.2 0.2) to those obtained by prioritizing each layer using a restart probability of 0.6 while keeping the others at 0.1. (**C**) Zoomed-in view of the boxplots shown in panel A. (**D**) Zoomed-in view of the boxplots shown in panel B.
